# Cohort profile update–overview of over 35 years of research in the Dortmund Nutritional and Anthropometric Longitudinally Designed (DONALD) study

**DOI:** 10.1007/s00394-023-03290-x

**Published:** 2023-12-27

**Authors:** Ines Perrar, Ute Alexy, Ute Nöthlings

**Affiliations:** Institute of Nutritional and Food Sciences-Nutritional Epidemiology, University of Bonn, Friedrich-Hirzebruch-Allee 7, 53115 Bonn, Germany

**Keywords:** DONALD, Cohort study, Children, Adolescence, Nutrition, Epidemiology

## Abstract

**Purpose:**

To provide an update on the cohort profile of the DOrtmund Nutritional and Anthropometric Longitudinally Designed (DONALD) study, including objectives, study design, methods and description of the comprehensive data pool, as well as to summarize the most important research findings of recent years.

**Methods:**

In 1985, the open (dynamic) cohort started to collect information on diet, growth, development, and metabolism of healthy children and adolescents in Dortmund, Germany. Detailed data are collected annually during infancy, childhood, and adolescence of the participants, including a 3-day weighed dietary record, a 24-h urine sample, anthropometric and medical examinations as well as interviews on lifestyle.

**Results:**

Even if the basic examination modules have not changed since the start over 35 years ago, the DONALD study has been continuously further developed by introducing new modules. As such, participants are also invited for follow-up examinations during adulthood since 2005, including an additional fasting blood withdrawal. Overall, 2375 (♂: 1177; ♀: 1198) participants were recruited in the DONALD study between 1985 and 2022. Data from ~ 30,700 anthropometric measurements, ~ 19,200 dietary records, ~ 10,600 24-h urine and ~ 1300 blood samples are available from an observation period of over 35 years.

**Conclusion:**

The DONALD study provides a large data pool for longitudinal studies on nutrition, growth, and health in childhood and adolescence, its impact on the development of diseases in early adult life as well as dietary intake trends over more than three decades.

**Supplementary Information:**

The online version contains supplementary material available at 10.1007/s00394-023-03290-x.

## Introduction

It has long been recognized that adequate nutrition is essential for physical and mental development in childhood and adolescence [1]. In addition, childhood and adolescence represent important periods in life for shaping dietary and lifestyle patterns, which may track into adulthood [2]. Nutrition across the life course of childhood, adolescence and early adulthood also affects health outcomes, i.e., the development of non-communicable diseases later in life [3–5]. However, many research questions about the specific role of nutritional, metabolic, and lifestyle factors during childhood on healthy growth as well as their impact on the development of diseases in later life are still unresolved. This may be due to the fact, that in researching the impact of nutrition on the growing organism, continuous and heterogeneous changes of the body and its functions in the course of development from infancy to adulthood pose a particular difficulty for data collection and analyses [6]. Therefore, comprehensive and close-meshed long-term studies with prospective and repeated detailed data collection on nutrition, metabolism, development, and growth among children and adolescents are essential to gain scientifically proven knowledge about nutrition and health associations. Findings from these studies may furthermore help to prevent chronic diseases in populations by providing research-based information for the development of evidence-based food-based dietary guidelines and further public health measures.

Against this background, the DOrtmund Nutritional and Anthropometric Longitudinally Designed (DONALD) study was designed more than 35 years ago as an open (dynamic) cohort study, in Dortmund, Germany. The study was first described in an international journal in 2004 [6]. A second description followed in 2012, albeit in German only [7]. Due to the dynamic design of the DONALD study, methods are continuously expanded. Hence, the aim of the current paper is to provide a detailed study update, including the description of study design, comprehensive methodology as well as available data and recent study results, to outline its great potential for addressing current research topics in nutritional epidemiology.

## Study objectives

The DONALD study was initiated in 1985 at the former German Research Institute of Child Nutrition Dortmund (Forschungsinstitut für Kinderernährung Dortmund, FKE). Since 2012, the study has been affiliated to the Faculty of Agriculture at Bonn University and is coordinated at the Institute of Nutrition and Food Sciences.

The longitudinal study started to collect information on diet, growth, development, and metabolism of healthy children and adolescents in August 1985. Initially, the following main research objectives were defined:

- Analyses of interactions between nutrition, metabolism, development, and growth in healthy children
- Description of intra- and inter-individual as well as age and time trends in dietary intake and patterns
- Determination of nutritional needs in children and adolescents
- Development of metabolic reference data based on 24-h urinary excretion levels in healthy children and adolescents
- Provision of dietary intake data for specific exposure assessments [6]

Over the past 35 years, however, the focus has continuously been widened to enable the investigation of further complex and societally relevant epidemiological research questions. For example, since data collection has continued into participants’ adulthood, a further aim of the study is to investigate the impact of diet and lifestyle in childhood and adolescence on health outcomes in adulthood. In addition, with advances in omics-technologies, phenotypic and genotypic data have been expanded.

## Study design and methods

### Study recruitment

The DONALD study started with a cross-sectional sample of children and adolescents (~ 640 participants, > 2 years old) recruited in the first decade since 1985. In addition, since study start, 35–40 infants are newly recruited every year. The recruitment of participants is carried out unsystematically through maternity wards, pediatric practices, other pediatric institutions, personal contacts or recommendations from study participants, in the city of Dortmund and the surrounding communities. Eligible infants are healthy (i.e., no prevalent diseases affecting growth and/or diet), with parents willing to participate in a long-term study and with at least one parent having sufficient knowledge of the German language. To date, these inclusion criteria have not been changed.

### Study design

**Fig. 1:**
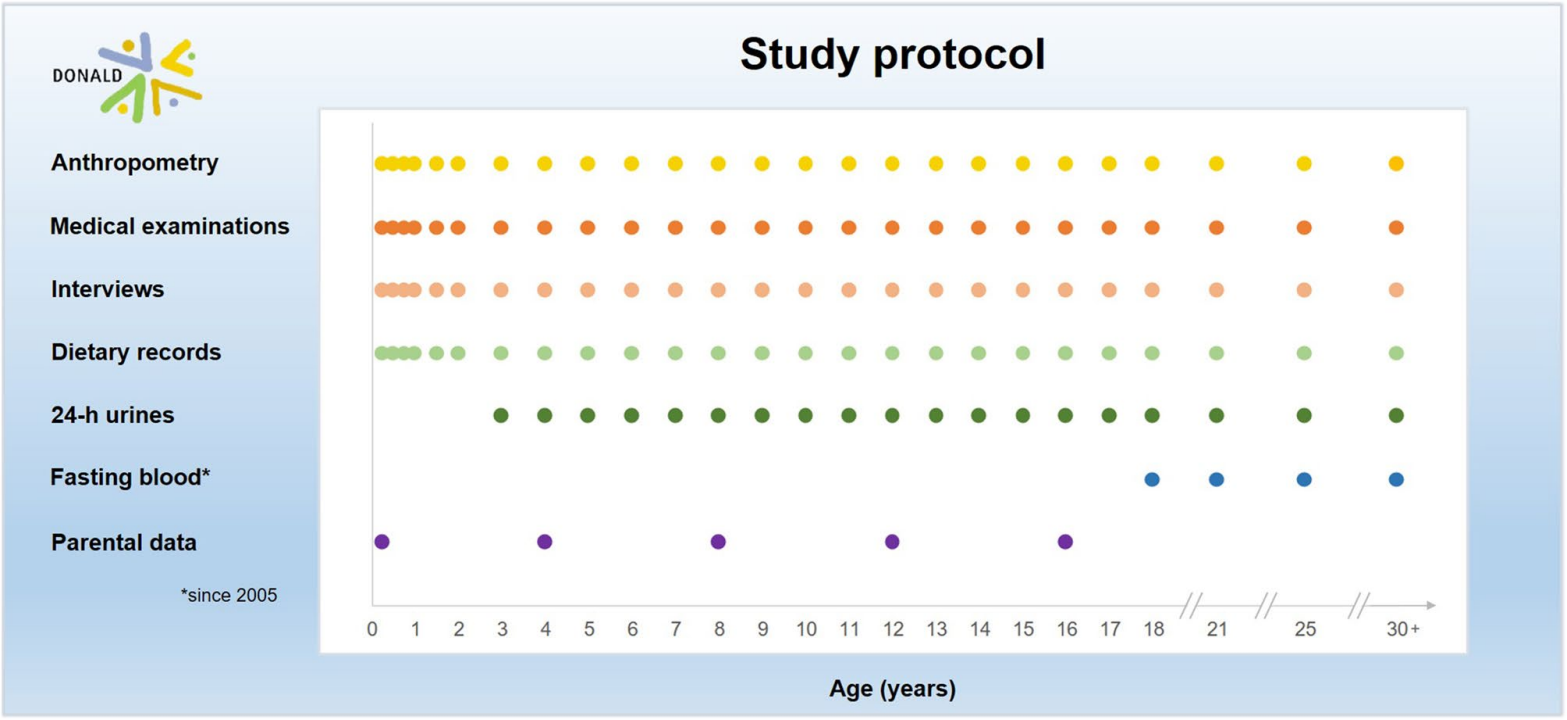
Study protocol of the DONALD study: data collection depending on the age (years) of the participants

According to the design of the DONALD study (Fig. 1), participants are examined for the first time at the age of 3 months. The participants return to the study center for three more visits in the first year of life, two in the second year and annually thereafter until young adulthood. Yearly examinations include 3-day weighed dietary records, anthropometric measurements, the collection of one 24-h urine sample (starting at age 3–4 years), medical examinations as well as interviews on lifestyle (e.g., physical activity, chronobiology, socioeconomic factors). Between 1986 and 2009, ~ 580 children and adolescents participated in a reduced program of the DONALD study without collecting dietary records and 24-h urines (Fig. S1). Participants were allocated to this reduced program, if they were not compliant to complete dietary records and/or 24-h urines. However, the frequency of study center visits for anthropometric and medical examination followed the study protocol.

The study is non-invasive until the age of 18 years. Since 2005, participants older than 18 years are also invited to provide a fasting blood sample. Parental examinations (anthropometric measurements, measurement of blood pressure, lifestyle interviews) take place every 4-years. The design of the DONALD study was approved by the Ethics Committee of the University of Bonn (project identification of the most recent version: 185/20), according to the guidelines of the Declaration of Helsinki. All examinations are performed with parental as well as from adolescents (16 years) onwards, with participants’ written consent.

### Data assessment

#### Anthropometrics

During the study center visit, trained nurses carry out detailed anthropometric measurements according to standardized procedures, using regularly calibrated instruments [8, 9]. Quality controls of the measurements are regularly conducted.

For the measurements, participants are wearing underwear only as well as no shoes. Since 1985, height or length (to 0.1 cm), weight (to 100 g), upper arm circumference (to 0.1 cm), as well as four skinfolds (biceps, triceps, subscapular and iliac skinfolds, to 0.1 mm) are measured at each examination. Among children < 2 years of age, recumbent length is measured. From the age of 2 years onwards, standing height is measured. Both are assessed using a digital stadiometer (Harpenden, Crymych, UK). Body weight is measured using an infant weighing scale (until 08/2018: Mettler PS 15; Mettler Toledo, Columbus, OH; from 08/2018 onwards: Seca 757; Seca Weighing and Measuring System, Germany) as well as an electronic scale (Seca 920; from 02/2006 onwards Seca 701; Seca Weighing and Measuring System, Germany) among older participants. To estimate body composition, [10–12], four skinfolds (biceps, triceps, iliacal, and supradings) are measured on the right side of the body using a skinfold calliper (Holtain Ltd, Croswell, Dyfed, UK), duplicate measurements are taken since 2006. Head circumference (accurate to 0.1 cm) has been assessed at every examination since 1985, from the first visit to age 5 as well as at ages 18 and 21, using diameter measuring tapes (until 09/2012: Circumference Diameter Tape, Chasmors, London, UK; from 09/2012 onwards: Diameter Measuring Tape, Richter, Germany). In addition, since 1985, sitting height (to 0.1 cm) is recorded among participants ≥ 3 years at each visit using a sitting height measuring device (custom-made for the DONALD study).

Since 2005, from the age of 3 years onwards, hip (around the widest portion of the buttocks) and waist (at the midpoint between lower rib and iliac crest) circumference (to 0.1 cm) are assessed. Abdominal circumference (to 0.1 cm) is been measured since 1985 at every visit to the study center, from study entry until 2 years of age. Measurement of upper and lower leg circumference was performed at each visit/every age of the participants between 1985 and 2006. Since 2006, these measurements are taken only at the first visit as well as at the visits at age 5, 10, 15, and 18–25.

Until 2004, anthropometric measurements were taken every 6 months during puberty (girls: 8–14 years, boys: 10–16 years) to reflect rapid anthropometric changes during this period.

#### Medical examination

At each study center visit, trained physicians conduct a medical examination. Furthermore, information about the current and past (related to the time period since the last visit) health status of the participant as well as the age-appropriate physical development are collected. A detailed anamnesis interview inquired among others chronic diseases that are documented as ICD codes. Other examinations include measurements of blood pressure, intima-media thickness of the carotid artery and thyroid volume.

The measurement of systolic and diastolic blood pressure is usually performed at regular time intervals from the age of 4 (until 2004) or 6 (since October 2004) years onwards by trained nurses, according to standard procedures (participant in seated position with legs uncrossed, feet at the floor, using the right arm, arm supported at heart level) [13, 14]. Until 1994, a random zero sphygmomanometer (Random Zero Sphygmanometer 77,075, Hawksley & Sons, UK) is used for the measurements, whereas a standard mercury sphygmomanometer (Sphygmomanometer Mercury 300, Speidel & Keller, Germany) was used between 1994 and 2020. Different cuff sizes are used according to arm circumferences. At each measurement occasion, two consecutive blood pressure readings are taken after a rest of ~ 5 min. Since 2020, a digital sphygmomanometer (Mindray VS-600, Mindray, Germany) is used.

Since 2008, intima-media thickness is measured every 2 years in schoolchildren (11–17 years) as well as during adulthood. For this, vascular condition of the left and right common carotid artery is determined by high-resolution ultrasonography (Until 2016: Mindray DP3300, Mindray Bio‐Medical Electronics Co., Ltd., Shenzhen, China; since 2016: Sonoscape S11, Sonoscape Co. Ltd., Shenzhen, China). Measurements are taken after a 10-min resting period in participants in a supine position, head slightly to the right or left according to a standard procedure [15–17].

Since 2012, the thyroid volume has also been determined by ultrasonography under the same conditions as the intima-media examination. Data are collected from children, every 2 years between 7 and 17 years, as well as at each visit during adulthood. Thyroid-lobe height, width, and depth were measured. From these data, thyroid volume is calculated by the formula of Brunn et al. [18].

#### Dietary assessment

Paper-based 3-day weighed dietary records are used to collect information on food and nutrient intake from all participants from infancy to adulthood. To estimate breast milk quantities by test-weighing [19] during infancy, mothers are provided a baby scale (Soehnle multina 8300, Soehnle, Germany) to weigh the child before and after each feeding to the nearest 10 g [20].

From the start of complementary feeding onwards, all foods and beverages consumed by the participant, as well as leftovers, are weighed and recorded over 3 consecutive days to the nearest 1 g by the parents or, later on, by participants themselves. If there is no digital scale in the household, families are provided with a digital scale (currently WEDO Digi 2000, WEDO, Germany) and are instructed by a dietician on how to use it for weighing. If exact weighing is not possible, e.g., when eating out-of-home meals or snacks, household measures (e.g., spoons, cups) are allowed for semiquantitative recording.

The beginning of the 3-day dietary recording is chosen by the participant themselves usually within 8 weeks after the study center visit. Requested information includes name of the food or drink, date, time and location of eating, as well as recipes (ingredients and preparation). The participants are asked to describe the food as detailed as possible, e.g., to also record the fat content of dairy products or bread with iodized salt. For commercial food items, the packages or the food labels are collected by the participants and information on the packaging (name, manufacturer, brand, labeled nutrients, list of ingredients) are added to the dietary record data. Medication and dietary supplement use are also recorded. After the 3 days of recording, a trained dietician picks up all materials at the family’s home or picture of packaging are collected via an online messenger. The dietician crosschecks the dietary record using standard questions (e.g., on brand, manufacturer, homemade recipes, preparation method, type of table salt) in a short interview/phone call with the participant. Missing information can, thus, be added. In addition, it is inquired whether unusual events during the recording days might have changed the habitual dietary behavior.

All recorded food items are coded and linked to the continuously updated in-house nutrient database „LEBensmittelTABelle“ (food table, LEBTAB [21]). LEBTAB was developed during the course of the DONALD study and contains detailed data (energy and 72 nutrients; version III) on all basic (e.g., apple or milk) and commercial composite foods (i.e., infant formula, commercial complementary food as well as ready-to-eat-meals/-products, fortified food, food for dietetic purpose, and nutrient supplements), which were recorded by DONALD participants. Data entries for basic foods are based mainly on the German standard food table “Bundeslebensmittelschlüssel“ (BLS; version 3.02; https://www.blsdb.de/). Missing food items are complemented by data from other national food tables, e.g., by the U.S. Department of Agriculture (USDA) [22]. Energy and nutrient contents of composite foods are estimated by recipe simulation using labeled ingredients and declared nutrient contents taking into account nutrient fortification. In addition, food additives and flavorings have been recorded qualitatively in the estimated recipes since 2004.

Energy and nutrient data in LEBTAB refer to the raw food item. To take the loss of vitamins during food preparation into account, corresponding subtractions are made based on average nutrient losses during preparation as published by the German Nutrition Society [23]. The 3-day weighed records have been validated against the biomarker of 24-h urinary total nitrogen excretion [24], which showed that protein intake in children and adolescents can be estimated with acceptable validity by weighed dietary records.

Due to the longitudinal character of the DONALD study, recipe modifications or reformulations of commercial food products by the manufacturers are updated continuously. Therefore, each modified product receives a new entry in the nutrient database, while the entry for the product with the old recipe is marked and retained in the database. Until the end of 2022, LEBTAB contains more than 17,000 entries, i.e., ~ 1500 basic foods, ~ 15,700 composite foods (including ~ 4900 fortified products), and ~ 470 medication (e.g., antibiotics, analgesics) and supplements (e.g., nutrients, botanicals). In addition, as part of specific research projects, LEBTAB or at least parts of the included food codes, is regularly expanded to additional nutritional information (e.g., whole grain [25], glycemic index [25], isoflavones [26] or anthocyanin [27], or free sugar [28]).

#### Interviews

##### Early life factors

At the first visit, each child’s birth characteristics are retrieved from the German standardized pregnancy document called “Mutterpass”. This includes information on pregnancy duration, type of birth (vaginal or cesarean), birth weight, birth size, head circumference of the child as well as the APGAR score at 5 and 10 min after birth [29]. In addition, a detailed interview on pregnancy is conducted with the mother. To be able to calculate weight gain during pregnancy, maternal body weight at the beginning and the end of pregnancy is inquired about. Furthermore, information on pregnancy symptoms and illnesses is collected, e.g., occurrence and duration of nausea as well as feeling of fullness/reflux and its consequences for eating behavior, iron deficiency, high blood pressure, diabetes or kidney diseases. Questions on maternal lifestyle during pregnancy concern smoking behavior before and during pregnancy, including number of cigarettes/day; alcohol consumption and whether, how long and until which month of pregnancy the mother did exercise. In addition, mothers are asked about their intake of dietary supplements during pregnancy. A detailed questionnaire on breastfeeding behavior is completed at all visits during infancy. Thus, data on the duration of full and partial breastfeeding are collected. In addition, mothers are asked whether they had planned to breastfeed before the birth of their child and the reason for stopping breastfeeding. Formula feeding as well as the start of complementary feeding are also documented.

##### Physical activity

In DONALD, a standardized questionnaire based on the Adolescent Physical Activity Recall Questionnaires [30] and questions from the German Health Interview and Examination Survey for Children and Adolescents (KiGGS) [31] is used to assess physical activity as well as sedentary behavior since 2004. Questions on physical activity include the duration and frequency of organized (e.g., club sport, gym) and unorganized sports (e.g., playing football with friends, cycling) as well as how the participant usually gets to school (e.g., by car, bus, bike or walking). Furthermore, duration and frequency of sedentary activities, i.e., watching television or reading a book, are asked.

Since June 2004, muscle function/physical performance tests have been used every 2 years from the age of 6 and on every occasion in adulthood with the help of a Ground Reaction Force Platform (GRFP; NOVOTEC Medical GmbH). In addition, since 2004 on the same occasions, muscle strength/grip strength is determined using a dynamometer (Jamar Jackson, MI, 49203 U.S.A.) on the non-dominant hand (participant in an upright-seated position and feet at the floor).

##### Chronobiology

Since 2014, chronotype of DONALD participants during adolescence has been assessed with the Munich Chronotype Questionnaire (MCTQ). The MCTQ includes information on sleep duration and bed times, separately for free and for school/working days, and therefore allows to calculate chronotypes and social jetlag according to Roenneberg [32, 33].

##### Further interviews

At each visit, further personal interviews with participants’ parents and/or older participants themselves are conducted by the study pediatricians, including inquiring about data on childcare, use of vitamin D and fluoride supplements and preventive medical services. From the age of 12 onwards, participants are also regularly asked to complete questionnaires on smoking habits, intake of alcoholic beverages, and restrictive dietary behavior in absence of their parents.

#### Urine sampling and analysis

From 3 or 4 years onwards, 24-h urine is collected once annually preferably on the third day of dietary recording. Parents as well as older children receive in-person and written instructions on how to collect complete 24-h urine samples according to a standardized procedure. The collection starts after voiding of the bladder upon getting up in the morning. This micturition is completely discarded. The time of this micturition is recorded and defines the start of the 24-h collection, which ends with the first micturition on the following day. During the collection period, the participants store micturitions immediately in preservative-free, Extran-cleaned (Extran, MA03; Merck, Darmstadt, Germany) 1-L plastic containers at less than − 12 °C before transfer to the study center. The exact times of micturition as well as possible comments on the collection are recorded on a predefined protocol sheet. The dietician picks up the 24-h urine samples together with the dietary record, checks plausibility of the collection protocol, inquires parents and/or participants about the completeness of the urine samples, and adds this information to the protocol sheet if necessary. After the transfer to the study center, the containers are stored at − 22 °C until analysis and subsequent biobanking. For routine laboratory measures and aliquotation, the samples are thawed and stirred. Urine volume, creatinine, urea, sodium, potassium, osmolality, and pH value are analyzed in the DONALD study laboratory. Subsequently, five aliquots of 20 ml each from each 24-h urine are stored at − 22 °C in the DONALD biobank for further research. Since the beginning of the DONALD study in 1985, over 11,000 24-h urine samples were collected and added to the biobank (Table 2).

Until 2019, for younger participants (0 − < 3 years), a spot urine is collected at the study center visit using a urine bag (Braun AG, Germany). After creatinine concentration measurements, samples were stored preservative free at − 22 °C in the urine biobank of the DONALD study (*n* = 1134).

#### Blood withdrawal and analyses

Since 2005, participants over the age of 18 have been asked to provide a venous fasting blood sample. Blood samples are centrifuged directly at 4 °C within 15 min (3100 U/ min). The blood samples are routinely analyzed to determine among others blood cell levels (e.g., leucocytes, thrombocytes, erythrocytes), hemoglobin, hematocrit, liver enzymes (GOT, GPT, GGT), cholesterol and blood lipid levels (e.g., total cholesterol, LDL, HDL), triglycerides, blood sugar, iron levels and thyroid parameters (TSH, fT3, fT4). Aliquots of serum, plasma (citrate, EDTA) and buffy coat (citrate, EDTA) samples (each 500 µl) are stored at − 80 °C in the DONALD biobank for further research.

#### Parental data

The study nurses collect maternal and/or paternal anthropometric (body weight and height, skinfolds, hip and waist circumference) as well as medical data (blood pressure) every 4 years using the same methodology and equipment as for the participants. In addition, parents are asked about previous chronic diseases (including hypertension, hyperlipidemia, diabetes, fatty liver disease), the intake of medication as well as diagnosed allergies. Furthermore, data on parental lifestyle (e.g., smoking in household, physical activity, specific dietary practices) and socioeconomic status (e.g., parental education and employment) are collected from both parents.

During adulthood (from the age of 21 onwards), the participants themselves are asked about their parents’ data, e.g., chronic diseases as well as if necessary, year of mortality and the cause of death.

#### Additional data collection

##### Additional modules

In addition to the basic protocol of the DONALD study, further modules among subgroups are established within the framework of specific projects (Table S1). Methods are described in short as follows.

Stool samples were collected between 2017 and 2018 in the HEALTHMARK (metabolic HEALTH through nutrition, microbiota and tryptophan bioMARKers) project from adult participants (≥ 18 years) to be able to explore gut microbiome-related research questions. Finally, microbiome data based on 16S ribosomal RNA sequencing of *n* = 128 individuals (77 double and 51 single stool samples) are available [34]. Currently, i.e., since 2023, further stool samples have been collected from the adult participants as part of the PerMiCCion (PERsonalized MIcrobiome-Based Approaches to Early Onset Colorectal Cancer PreventION, Diagnosis and Management) project (http://www.permiccion.de/).

A variety of additional modules were made possible within the framework of the nutrition research competence cluster Diet Body Brain (DietBB; www.diet-body-brain.de), including the collection of genetics, data on fluid intelligence and personality-related characteristics accelerometer. Between 2015 and 2022, saliva samples were used for DNA extraction and one time collected among children and adolescents between 6 and 17 years (*n* = 170). Among adult participants, blood samples (buffy coats (*n* = 440) and PaxGene samples (*n* = 287)) are used to analyze DNA and RNA. These data allow the investigation of associations between nutrition, genetics, and health.

To investigate associations between personality-related characteristics and nutrition or anthropometry as part of DietBB (2016–2020), individual personality data were collected. For this, game-based experiments were conducted. Among 5–6 year olds (*n* = 126; until June 2016 also 4 year olds), delay of gratification was assessed, via Stanford marshmallow experiment [35]. To assess economic preferences, experiments on time, social, and risk preferences [36] were performed among 7–9 year olds (*n* = 168). In addition, validated questionnaires on personality by Decker et al. [36] were used, which include personality questions on the consumption of sweets (*n* = 125; age 5–6 years, until June 2016 also 4 year olds) or pocket money (*n* = 167; age 7–9 years).

Fluid intelligence was assessed using the Culture Fair Intelligence (CFT) 1-R (*n* = 89) [37] or the CFT 20-R test (8.5–18 years *n* = 139; > 18 years *n* = 99) [38] among a DONALD subgroup in 2017–2018. The cognition test was carried out once per participant. CFT 1-R was used among participants between 5 and < 8.5 years, CFT 20-R was used among participants from 8.5 years onwards. Both tests were developed language-free and figure-based to minimize cultural or social disadvantages or advantages [37, 38].

Accelerometer (CentrePoint Insight Watch from the company ActiGraph) data have been collected from 2020 until 2022 to determine physical activity and sleep characteristics (quality and duration) among participants between 6–18 years (*n* = 127) and > 18 years (*n* = 36).

In a further additional module in 1989–1999, a single peripheral quantitative computed tomography (pQCT) analysis of the forearm was undertaken in 371 participants aged 6–18 years. Details of these data collection and measurements can be found in numerous publications (among others: [39–41]).

##### Additional biosample analyses

Additional analyses of biosamples (urine and blood) are carried out within the framework of specific research projects, partly in cooperating laboratories. According to the respective research question, project-specific metabolic parameters were measured in urine samples among subgroups of the study. These include among others metabolomics (e.g., described in [42]), hippuric acid as biomarkers for flavonoid intake (e.g., described in [43]), fructose/sucrose excretion as biomarkers for total sugar intake (described in [44, 45]) and nutritionally and metabolically relevant hormone metabolites, e.g., adrenal androgens, glucocorticoids (described in [46, 47]). Similar to these urinary analyses, additional blood analyses were conducted within specific research projects, e.g., untargeted metabolomics or several serum (e.g., hormones (IGF-1, leptin), inflammatory markers (IL-18, E-selectin, IL1-ra, hs-CRP) and EDTA plasma parameters (e.g., hormones (insulin, adiponectin, fT3, fT4, TSH), inflammatory markers (IL-6, IL-18, hs-CRP, sICAM-1, omentin, fetuin-A), triglycerides, liver enzymes (GOT, GGT, GPT, ALT, AST)). Data from blood sample analyses are used primarily to determine metabolic health during adulthood, e.g., type II diabetes [3, 48], non-alcoholic fatty liver [4, 49] or cardiometabolic diseases [5, 50].

### Data availability

The DONALD study aims to make research data available in accordance to the FAIR principles [51], and within the framework of data protection regulations. Research data can be made available upon request to epi@uni-bonn.de.

The study has been registered at the German Clinical Trials Register (DRKS00029092) and metadata of the study is published in the metadata portal for observational studies in Nutritional Epidemiology, which participated in the INTIMIC project (https://mica.mdc-berlin.de/study/donald) as well as in the NFDI4Health metadata portal (https://csh.nfdi4health.de/resource/45).

## Results

### DONALD cohort

**Fig. 2:**
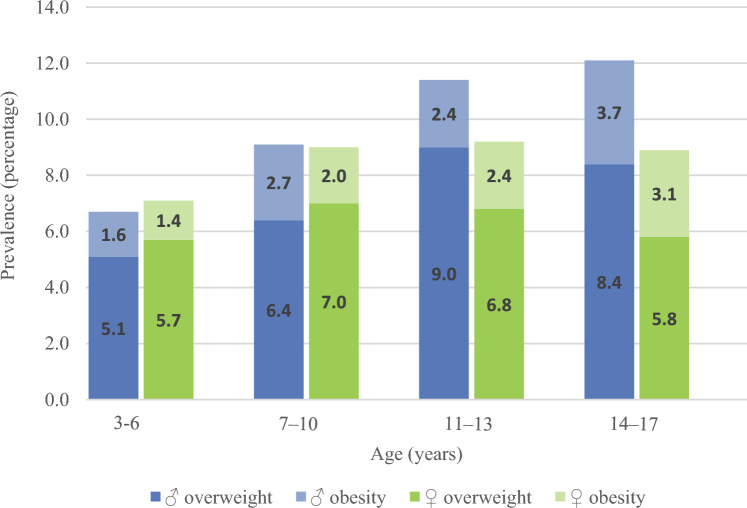
Prevalence for overweight and obesity among male (3–6: *N* = 2785, *n* = 802; 7–10: *N* = 2771, *n* = 762; 11–13: *N* = 2501, *n* = 630; 14–17: *N* = 2512, *n* = 593) and female (3–6: *N* = 2676, *n* = 801; 7–10: *N* = 3182, *n* = 725, 11–13: *N* = 2483, *n* = 645; 14–17: *N* = 1876, *n* = 527) participants of the DONALD study in different age groups. Prevalence for overweight (BMI-SDS > 90th percentile and < 97th percentile) and obesity (BMI-SDS > 97th percentile) was calculated based on the German reference percentiles for children and adolescents by Kromeyer–Hauschild [52]

Overall, 2375 (♂: 1177; ♀: 1198) participants have been recruited between 1985 and 2021. Study sample characteristics are shown in Table 1. Compared to the general population in Germany, participating families in DONALD are characterized by a rather high socioeconomic status (Table 1, [6]). The high socioeconomic status is also reflected in the prevalence of overweight and obesity in DONALD (Fig. 2).

**Table 1:** Sample characteristics of the DONALD population (1985 – 2022)

	Total	Male	Female
n_total_^1^	2375	1177	1198
1985–1994^1^	1275	626	649
1995–2004	452	213	239
2005–2014	372	199	173
2015–2022	276	139	137

		Total available data (*n*)	Frequency (*n* (%))	Total available data (*n*)	Frequency (*n* (%))	Total available data (*n*)	Frequency (*n* (%))
*Early life factors*							
Birth weight ≥ 3000 g		2222	1853 (83.4)	1100	945 (86.0)	1122	908 (80.9)
Birth length ≥ 50 cm		2216	1834 (82.8)	1096	940 (85.8)	1120	894 (79.8)
Gestational age ≥ 40 weeks		2181	1345 (61.7)	1078	660 (61.2)	1103	685 (62.1)
Full breastfeeding ≥ 4 months		1871	1043 (55.8)	922	512 (55.5)	949	418 (44.1)
*Socioeconomic factors*^2^							
Maternal overweight^3^		2025	625 (30.9)	998	318 (31.9)	1027	307 (29.9)
Paternal overweight^3^		1660	941 (56.7)	826	467 (56.5)	834	474 (56.8)
Maternal high educational status^4^		2335	1378 (59.0)	1161	694 (59.8)	1174	694 (58.3)
Paternal high educational status^4^		2335	1404 (60.1)	1161	708 (61.0)	1174	696 (59.3)
Maternal employment		1810	498 (27.5)	890	243 (27.3)	920	255 (27.7)
Paternal employment		1815	1754 (96.6)	910	881 (96.8)	905	873 (96.5)
Smoker in household		1600	423 (26.4)	797	206 (25.9)	803	217 (27.0)

Values are total numbers of participants from whom data are available or absolute and relative frequencies of the respective listed characteristic

^1^In the first study years, 640 participants > 2 years were recruited

^2^First available parental assessment

^3^BMI ≥ 25 kg/m^2^

^4^≥ 12 years of schooling

The data pool of the DONALD study, i.e., numbers of 3-day weighed dietary records, anthropometric measurements, 24-h urine and blood samples in the DONALD study (1985–2021) are presented in Table 2, stratified by age and sex groups. In total, data from ~ 30,700 anthropometric measurements, ~ 19,200 dietary records, ~ 10,600 24-h urine, and ~ 1300 blood samples are available. Although the study protocol requires data to be collected from infants, children, and adolescents at least once a year (Fig. 1), the assessments are based on a voluntary basis. Therefore, and due to the very time-consuming data collection, data are not available from each participants for all assessments at all ages and the total number of assessments differ. In addition, the reduced program (Fig. S1) of the study as well as additional measurements every 6 months during puberty between 1985 and 2004 in the cohort, led to the relatively high number of anthropometric examinations. Even though the number of participants is continuously increasing, the number of collected records and urine samples per year is decreasing over the last two decades (data not shown). Socioeconomic parameters of families also changed over the study years. The proportion of parents with a high level of education (≥ 12 years of schooling) increased, as did the proportion of mothers who were employed (data not shown).

**Table 2:** Number of 3-day weighed dietary records, anthropometric measurements, 24-h urine and blood samples collected between 1985 and 2022 in the DONALD study, stratified by age and sex groups

Age (years)	Total				Male				Female			
	Dietary records	Anthropometric measurements	24-h urine samples^1^	Blood samples^2^	Dietary records	Anthropometric measurements	24-h urine samples^1^	Blood samples^2^	Dietary records	Anthropometric measurements	24-h urine samples^1^	Blood samples^2^
Total	19,204 (100)	30,734 (100)	10,585 (100)	1,333 (100)	9727 (50.7)	15,410 (50.1)	5377 (50.8)	595 (44.6)	9477 (49.3)	15,324 (49.9)	5208 (49.2)	738 (55.4)
≤ 2	6773 (34.8)	8529 (27.8)	–	–	3291 (34.9)	4256 (27.6)	–	–	3282 (34.6)	4273 (27.8)	–	–
3–7	4867 (25.3)	6676 (21.7)	3501 (33.1)	–	2497 (25.7)	3422 (22.2)	1770 (32.9)	–	2370 (25.0)	3254 (21.2)	1731 (33.2)	–
8–12	3859 (20.1)	8136 (26.5)	3510 (33.2)	–	1984 (20.4)	3832 (24.9)	1823 (33.8)	–	1875 (19.8)	4304 (28.1)	1687 (32.4)	–
13–17	2756 (14.4)	5395 (17.6)	2573 (24.3)	–	1424 (14.6)	3008 (19.5)	1362 (25.3)	–	1332 (14.1)	2387 (15.6)	1211 (23.3)	–
≥ 18	1049 (5.5)	1998 (6.5)	1001 (9.5)	1,333 (100)	431 (4.4)	892 (5.8)	422 (7.9)	595 (44.6)	618 (6.5)	1106 (7.2)	579 (11.1)	738 (55.4)

Values are absolute and relative frequencies

^1^Urine samples were collected from 3–4 years onwards

^2^Blood samples were collected among adult participants (≥ 18) only

### Exemplary results of recent years

Since 1985, the DONALD study has led to numerous nutritional, growth, and metabolism-related publications in national and international journals. Exemplary results of the DONALD study in recent years are briefly described in Table 3. Results of the DONALD studies are incorporated into the work of various national and international professional societies. For example, results of the project on complementary feeding [53, 54] were taken up by the Nutrition Commission of the European Society for Paediatric Gastroenterology, Hepatology, and Nutrition (ESPGHAN) [55] as well as by the British Nutrition Foundation [56], in their position papers on complementary feeding. As the DONALD study was the first study, which provided data on free sugar intake among children and adolescents in Germany [28], these were included in the consensus paper by the German Obesity Society, German Diabetes Society and German Nutrition Society on quantitative recommendations for sugar intake in Germany [57]. In addition, the European Food Safety Authority also refers repeatedly to different data from the DONALD study in its scientific opinions and recommendations (e.g., [58–60]).

**Table 3:** Results from selected research projects of the DONALD study in recent years

Research topic	Main results
Sustainable diet	In a comprehensive analysis, associations of adherence to the EAT-Lancet reference diet with nutritional, anthropometric, and ecological sustainability parameters were investigated. Adherence to the EAT-Lancet reference diet was associated with favorable nutritional characteristics, e.g., lower added sugar and higher fiber intake and reduced environmental impact (greenhouse-gas emissions and land use) among adolescents (≥ 15 years) [61]. Adherence to the diet during adolescence also had a positive impact on anthropometric characteristics, e.g., BMI in early adulthood (≥ 18 years), but not on other cardiometabolic risk markers [61].
Gut microbiome	A recent paper investigated whether long-term energy, carbohydrate, fiber, protein, or fat intake from infancy to late adolescence has an impact on gut microbiota composition later in life among DONALD participants. The examination showed that long-term carbohydrate intake from infancy to late adolescence (1–18 years) is associated with gut microbiota composition in young adulthood (≥ 18 years) [34]. Subsequently, a prospective association between the composition of the gut microbiome and cognitive performance in healthy young adults (≥ 18 years) was observed [62].
Metabolomics	DONALD also contributes to basic metabolomics-related epidemiologic research questions. Among others, overweight was related to gut microbiome-derived indole-3-acetic acid and several amino acids, including some gut microbiome-derived metabolites were associated with systemic inflammation [42], sexual dimorphism in the association of urine metabolome and body composition was observed among adolescents. Significant associations of urine metabolites with body mass index and body fat were only found among male adolescents (16–18 years) [63]. Furthermore, a reflection of habitual food group intake (processed meat, eggs, vegetables) by single metabolites in urine but not in blood was observed [64]. Body composition- and food-related metabolites were also linked to cardiometabolic risk markers [65]. Finally, habitual physical activity is also associated with metabolite patterns [66].
Healthy lifestyle	Using a Lifestyle score, including diet, physical activity, sedentary behavior, sleep duration and/or BMI-SDS, the association between lifestyle during adolescence and the development of diseases in later life was studied. In this examinations, a higher lifestyle score during adolescence (9–16 years) was inversely associated with body composition [5] and fatty liver indices [4] in adulthood (≥ 18 years). Initial research on cognition in DONALD showed a positive association of healthy lifestyle with fluid intelligence among participants > 8.5 years [67]. Especially physical inactivity seemed to play a prominent role in this context, since increasing hours of sedentary behavior was associated with a lower fluid intelligence score.
Iodine status	Using iodine excretion of DONALD participants (6–12 years) in 24-h urines (gold standard), the rough estimates of iodine status using spontaneous urine samples in the representative surveys, KiGGS and the German health interview and examination survey for adults (Studie zur Gesundheit Erwachsener in Deutschland, DEGS), of the Robert Koch Institute (RKI) were regularly complemented or confirmed [68, 69]. Recent trend analyses [69] showed a decrease in iodine intake according to excretion levels in 24-h urines since 2012 among children (6–12 years), while sodium excretion increased. In addition, in cooperation with the RKI, detailed studies were conducted on changes in thyroid-stimulating hormone (TSH) depending on the iodine status [70]. The investigation showed that smaller thyroid volume is associated with higher normal TSH levels among thyroid-healthy children, which calls into question the common practice of primarily TSH-derived hypothyroidism diagnostics [70].
Chronotype, circadian eating patterns, social jetlag	An examination regarding the chronobiology of the DONALD participants showed differences in energy intake depending on the chronotype [71]. Adolescents (10–18 years) with a later chronotype consumed a lower proportion of their total daily energy intake before 11 a.m. compared to adolescents with an earlier chronotype [71]. This was due to a higher likelihood of breakfast skipping among later chronotypes [71]. In addition, adolescents with a later chronotype showed a larger energy intake after 6 p.m [71]. Investigations of the association between circadian eating patterns and long-term health showed that habitual low-fat and high-carbohydrate breakfast among pre-(3–4 years) and primary school (7–8 years) children may contribute to an increase of fat mass index in early adolescence (10–11 years) [72]. In addition, eating less amounts of carbohydrates from sources with a high glycemic index in the evening during adolescence (9–16 years) may help to reduce the risk of type 2 diabetes in adulthood (≥ 18 years) [48]. Furthermore, it is currently being investigated whether the individual chronotype or the so-called social jetlag [73] are associated with health outcomes.
Sugar intake	In a past project, detailed trends in total, added, and free sugar intake [28] as well as free sugar from food groups (e.g., sugar sweetened beverages, fruit juices, sweets) among children and adolescents were assessed [74]. Although analyses showed a decreasing time trend in total, added, and free sugar intake since 2005, especially since 2010, free sugar intake among the DONALD population exceeded the 10% of total energy intake limit, set by the WHO [75], across all age groups (3–18 years) and the whole observational period (1985–2016) [28]. The observed time trend among adolescents could be confirmed using urinary biomarkers for sugar intake [45]. In terms of age trends, it was shown that sugar intake of sugar sources differs depending on age: While free sugar intake from sugar sweetened beverages increased with age, the youngest children showed the highest intake of free sugar from juices as well as dairy products and primary school children showed the highest intake of free sugar from sweets [74].
Complementary foods	The DONALD study investigated whether the preparation method of complementary foods, i.e., home-cooked or commercial, plays a role in the development of individual food preferences [53, 54]. It was shown that a higher intake of commercial complementary foods is associated with lower vegetable intake in infancy [54]. Among male participants, an inverse association was observed also between the intake of commercial complementary foods and vegetable intake in preschool age [54]. A further evaluation showed that infants with higher intake levels of commercial complementary foods were more likely to have a high intake of added sugars from complementary foods as well as a higher overall added sugar intake [53]. In addition, intake of commercial complementary foods in infancy was positively associated with the intake of added sugars in preschool and primary school ages [53]. Since these associations were attenuated if the models were adjusted for overall added sugar intake in infancy, the overall added sugar intake among infancy, rather than the method of preparation, may have an impact on added sugar intake in later life [53]. Furthermore, the composition of commercial and homemade complementary foods was compared [76].

## Discussion

Due to its comprehensive and longitudinal design, the DONALD study offers the possibility to investigate prospectively a wide range of complex research questions on interaction of nutrition, growth, metabolism and long-term health. The study is furthermore characterized by the use of precise and close-meshed data collection methods, e.g., 3-day weighed dietary records. Furthermore, the 24-h urine allows metabolic insights and determination of biomarker for dietary intake. As the DONALD study is also collecting detailed data of participants during adulthood since 2005, it is further possible to investigate the impact of nutrition and lifestyle in phases, which are discussed as potentially critical for later disease risk, such as infancy/early childhood, primary school age or adolescence [77, 78]. This unique data collection enabled among others, the examination of long-term age and time trends on nutrition among children and adolescents in Germany over more than 3 decades from 1985 until today. Thus, results of the DONALD study contribute to the monitoring of habitual nutrient and food intake of children and adolescents, and therefore contribute to the basis for public health measures, including implementation in national and international nutrition recommendations or position papers [55–57].

To our knowledge, there is no comparable study in Germany or Europe with such a large number of repeated measurements among participants from infancy to adulthood and has such an extensive pool of nutritional, health and lifestyle data. There are some cohorts, such as IDEFICS (Identification and prevention of dietary and lifestyle-induced health effects in children and infants)/I.Family [79], KiGGs/Motorik-Modul Longitudinal Study [80], ALSPAC (Avon Longitudinal Study of Pregnancy and Childhood) [81], the Generation R [82] or Bogalusa Heart Study [83], which are larger and more representative. However, their study design includes fewer repeated measurements and/or does not cover detailed nutritional data or the entire observation in infancy, childhood, and adolescence, as well as continuing data collection in adulthood. To investigate research questions on the impact of nutrition and lifestyle in childhood and adolescence on the development of diseases in later life, prospective data on nutrition and its changes from infancy to adulthood are essential. The DONALD cohort can help to fill this research gap.

Although the design and methods of the DONALD study remained the same, some changes in the study population can be observed. The mean age of the study sample increases, as there are more adults invited to be examined. However, in evaluations of pediatric samples, an adjustment for age is mandatory. Second, the number of collected records and urine samples per year is decreasing over the last 2 decades. A decline in compliance with the study protocol was observed. At present, we can only speculate about the causes. One reason could be the more frequent occupation of both parents during the last decades in Germany, which leads to a greater time burden on families.

Nevertheless, the design of the study also leads to limitations. The detailed methodology and the elaborate design of the DONALD study result in a selected, non-representative study sample. Therefore, compared to general population in Germany, DONALD participants have a rather high socioeconomic status [6]. However, even in representative studies, it is difficult to recruit participants with a lower socioeconomic status for longitudinal observations. In addition, representative studies in Germany such as the nationwide SuSe study (“Stillen und Säuglingsernährung”) [84] or EsKiMo (“Ernährungsstudie als KiGGS-Modul”; substudy of a national survey named KiGGS (Kinder- und Jugendgesundheitssurveys)) [85, 86] show similar results, e.g., similar energy and nutrient intakes. The prevalence of overweight and obesity in DONALD is comparatively low, but similar to participants with a rather high socioeconomic status in KiGGS [87]. Furthermore, the study design leads to a rather small study sample. The statistical power for complex analyses requiring large numbers of cases is, therefore, rather low.

In summary, the DONALD study provides a large data pool for epidemiological research questions, which is continuously growing due to the dynamic, longitudinal design. In addition to descriptive evaluations, cross-sectional analyses and age trends, it is also possible to investigate long-term time trend analyses (> 35 years) as well as prospective exposure assessments. The study is, thus, essential for past and future monitoring of diet, growth, development, and health of children and adolescents in Germany and offers the potential to contribute to filling several research gaps.

### Supplementary Information

Below is the link to the electronic supplementary material.

Supplementary file1 (DOCX 28 KB)

## Data Availability

Data of the DONALD study are available upon request to epi@uni-bonn.de.

## References

[1] Martin HP (1973). Nutrition: its relationship to children’s physical, mental, and emotional development. Am J Clin Nutr.

[2] Mikkilä V, Räsänen L, Raitakari OT (2005). Consistent dietary patterns identified from childhood to adulthood: the cardiovascular risk in Young Finns Study. Br J Nutr.

[3] Penczynski KJ, Herder C, Krupp D (2019). Flavonoid intake from fruit and vegetables during adolescence is prospectively associated with a favourable risk factor profile for type 2 diabetes in early adulthood. Eur J Nutr.

[4] Schnermann ME, Schulz C-A, Perrar I (2022). A healthy lifestyle during adolescence was inversely associated with fatty liver indices in early adulthood—findings from the DONALD cohort study. Br J Nutr.

[5] Schnermann ME, Schulz C-A, Herder C (2021). A lifestyle pattern during adolescence is associated with cardiovascular risk markers in young adults: results from the DONALD cohort study. J Nutr Sci.

[6] Kroke A, Manz F, Kersting M (2004). The DONALD Study. History, current status and future perspectives. Eur J Nutr.

[7] Buyken AE, Alexy U, Kersting M (2012). Die DONALD Kohorte. Ein aktueller Überblick zu 25 Jahren Forschung im Rahmen der Dortmund Nutritional and Anthropometric Longitudinally Designed Study (The DONALD cohort. An updated overview on 25 years of research based on the Dortmund Nutritional and Anthropometric Longitudinally Designed study). Bundesgesundheitsblatt Gesundheitsforschung Gesundheitsschutz.

[8] Flügel B, Greil H, Sommer K (1986) Anthropologischer Atlas: Grundlagen und Daten; Deutsche Demokratische Republik, 1. Aufl. Verl. Tribüne, Berlin

[9] Lohman TG, Roche AF, Martorell R (1991). Anthropometric standardization reference manual.

[10] Durnin JV, Womersley J (1974). Body fat assessed from total body density and its estimation from skinfold thickness: measurements on 481 men and women aged from 16 to 72 years. Br J Nutr.

[11] Slaughter MH, Lohman TG, Boileau RA (1988). Skinfold equations for estimation of body fatness in children and youth. Hum Biol.

[12] Deurenberg P, Weststrate JA, Seidell JC (2007). Body mass index as a measure of body fatness: age- and sex-specific prediction formulas. Br J Nutr.

[13] Krupp D, Shi L, Remer T (2014). Longitudinal relationships between diet-dependent renal acid load and blood pressure development in healthy children. Kidney Int.

[14] Krupp D, Westhoff TH, Esche J (2018). Prospective relation of adolescent citrate excretion and net acid excretion capacity with blood pressure in young adulthood. Am J Physiol Renal Physiol.

[15] Nyasordzi J, Penczynski K, Remer T (2020). Early life factors and their relevance to intima-media thickness of the common carotid artery in early adulthood. PLoS One.

[16] Kassenärztliche Bundesvereinigung (2008) Vereinbarung von Qualitätssicherungsmaßnahmen nach § 135 Abs. 2 SGB V zur Ultraschalldiagnostik (Ultraschall-Vereinbarung). http://www.kbv.de/media/sp/Ultraschallvereinbarung.pdf. Accessed 29 Jun 2022

[17] (1996) Ultraschall-Kursbuch: Nach den Richtlinien der DEGUM und der KBV, 2., überarbeitete und erweiterte Auflage. Georg Thieme Verlag, Stuttgart

[18] Brunn J, Block U, Ruf G (1981). Volumetrie der Schilddrüsenlappen mittels Real-time-Sonographie (Volumetric analysis of thyroid lobes by real-time ultrasound (author's transl)). Dtsch Med Wochenschr.

[19] Butte NF, Garza C, Smith EO (1983). Evaluation of the deuterium dilution technique against the test-weighing procedure for the determination of breast milk intake. Am J Clin Nutr.

[20] Schoen S, Sichert-Hellert W, Kersting M (2009). Validation of energy requirement equations for estimation of breast milk consumption in infants. Public Health Nutr.

[21] Sichert-Hellert W, Kersting M, Chahda C (2007). German food composition database for dietary evaluations in children and adolescents. J Food Compos Anal.

[22] U.S. Department of Agriculture Food Composition. https://nal.usda.gov/legacy/fnic/food-composition. Accessed 07 Feb 2022

[23] Deutsche Gesellschaft für Ernährung (2015). D-A-CH Referenzwerte für die Nährstoffzufuhr.

[24] Bokhof B, Günther ALB, Berg-Beckhoff G (2010). Validation of protein intake assessed from weighed dietary records against protein estimated from 24 h urine samples in children, adolescents and young adults participating in the Dortmund Nutritional and Longitudinally Designed (DONALD) Study. Public Health Nutr.

[25] Goletzke J, Buyken AE, Joslowski G (2014). Increased intake of carbohydrates from sources with a higher glycemic index and lower consumption of whole grains during puberty are prospectively associated with higher IL-6 concentrations in younger adulthood among healthy individuals. J Nutr.

[26] Cheng G, Remer T, Prinz-Langenohl R (2010). Relation of isoflavones and fiber intake in childhood to the timing of puberty. Am J Clin Nutr.

[27] Drossard C, Bolzenius K, Kunz C (2013). Anthocyanins in the diet of children and adolescents: intake, sources and trends. Eur J Nutr.

[28] Perrar I, Schmitting S, Della Corte KW (2020). Age and time trends in sugar intake among children and adolescents: results from the DONALD study. Eur J Nutr.

[29] American Academy of Pediatrics Committee on Fetus and Newborn, American College of Obstetricians and Gynecologists Committee on Obstetric Practice (2015). The Apgar score. Pediatrics.

[30] Booth ML, Okely AD, Chey TN (2002). The reliability and validity of the Adolescent Physical Activity Recall Questionnaire. Med Sci Sports Exerc.

[31] Finger JD, Mensink GBM, Banzer W (2014). Physical activity, aerobic fitness and parental socio-economic position among adolescents: the German Health Interview and Examination Survey for Children and Adolescents 2003–2006 (KiGGS). Int J Behav Nutr Phys Act.

[32] Roenneberg T, Kuehnle T, Juda M (2007). Epidemiology of the human circadian clock. Sleep Med Rev.

[33] Roenneberg T, Allebrandt KV, Merrow M (2012). Social jetlag and obesity. Curr Biol.

[34] Oluwagbemigun K, O'Donovan AN, Berding K (2021). Long-term dietary intake from infancy to late adolescence is associated with gut microbiota composition in young adulthood. Am J Clin Nutr.

[35] Mischel W, Ebbesen EB (1970). Attention in delay of gratification. J Pers Soc Psychol.

[36] Deckers T, Falk A, Kosse F (2021). Socio-economic status and inequalities in children's IQ and economic preferences. J Polit Econ.

[37] Weiß RH, Osterland J (2012). CFT 1-R: Grundintelligenztest Skala 1.

[38] Weiß RH (2006). CFT 20-R: Grundintelligenztest Skala 2.

[39] Alexy U, Remer T, Manz F (2005). Long-term protein intake and dietary potential renal acid load are associated with bone modeling and remodeling at the proximal radius in healthy children. Am J Clin Nutr.

[40] Remer T, Manz F, Alexy U (2011). Long-term high urinary potential renal acid load and low nitrogen excretion predict reduced diaphyseal bone mass and bone size in children. J Clin Endocrinol Metab.

[41] Esche J, Johner S, Shi L (2016). Urinary citrate, an index of acid-base status, predicts bone strength in youths and fracture risk in adult females. J Clin Endocrinol Metab.

[42] Oluwagbemigun K, Anesi A, Ulaszewska M (2020). Longitudinal relationship of amino acids and indole metabolites with long-term body mass index and cardiometabolic risk markers in young individuals. Sci Rep.

[43] Penczynski KJ, Krupp D, Bring A (2017). Relative validation of 24-h urinary hippuric acid excretion as a biomarker for dietary flavonoid intake from fruit and vegetables in healthy adolescents. Eur J Nutr.

[44] Johner SA, Libuda L, Shi L (2010). Urinary fructose: a potential biomarker for dietary fructose intake in children. Eur J Clin Nutr.

[45] Perrar I, Gray N, Kuhnle GG (2020). Sugar intake among German adolescents: trends from 1990 to 2016 based on biomarker excretion in 24-h urine samples. Br J Nutr.

[46] Remer T, Shi L, Buyken AE (2010). Prepubertal adrenarchal androgens and animal protein intake independently and differentially influence pubertal timing. J Clin Endocrinol Metab.

[47] Shi L, Wudy SA, Buyken AE (2011). Prepubertal glucocorticoid status and pubertal timing. J Clin Endocrinol Metab.

[48] Diederichs T, Herder C, Roßbach S (2017). Carbohydrates from sources with a higher Glycemic Index during adolescence: is evening rather than morning intake relevant for risk markers of type 2 diabetes in young adulthood?. Nutrients.

[49] Perrar I, Buyken AE, Penczynski KJ (2021). Relevance of fructose intake in adolescence for fatty liver indices in young adulthood. Eur J Nutr.

[50] Oluwagbemigun K, Buyken AE, Alexy U (2019). Developmental trajectories of body mass index from childhood into late adolescence and subsequent late adolescence-young adulthood cardiometabolic risk markers. Cardiovasc Diabetol.

[51] Wilkinson MD, Dumontier M, Aalbersberg IJJ (2016). The FAIR Guiding Principles for scientific data management and stewardship. Sci Data.

[52] Kromeyer-Hauschild K, Wabitsch M, Kunze D (2001). Perzentile für den Body-mass-Index für das Kindes- und Jugendalter unter Heranziehung verschiedener deutscher Stichproben. Monatsschr Kinderheilkd.

[53] Foterek K, Buyken AE, Bolzenius K (2016). Commercial complementary food consumption is prospectively associated with added sugar intake in childhood. Br J Nutr.

[54] Foterek K, Hilbig A, Alexy U (2015). Associations between commercial complementary food consumption and fruit and vegetable intake in children. Results of the DONALD study. Appetite.

[55] Fewtrell M, Bronsky J, Campoy C (2017). Complementary feeding: a position paper by the European Society for Paediatric Gastroenterology, Hepatology, and Nutrition (ESPGHAN) Committee on Nutrition. J Pediatr Gastroenterol Nutr.

[56] Chambers L, Hetherington M, Cooke L (2016). Reaching consensus on a ‘vegetables first’ approach to complementary feeding. Nutr Bull.

[57] Ernst JB, Arens-Azevêdo U, Bitzer B, Bosy-Westphal A, de Zwaan M, Egert S, Fritsche A, Gerlach S, Hauner H, Heseker H, Koletzko B, Müller-Wieland D, Schulze M, Virmani K, Watzl B, Buyken AE for the German Obesity Society (DAG), German Diabetes Society (DDG) and German Nutrition Society (DGE) (2019). Quantitative recommendation on sugar intake in Germany. Short version of the consensus paper by the German Obesity Society (DAG), German Diabetes Society (DDG) and German Nutrition Society (DGE). Ernahrungs Umschau.

[58] Turck D, Bresson J-L, Burlingame B (2017). Scientific Opinion on the safety and suitability for use by infants of follow-on formulae with a protein content of at least 1.6 g/100 kcal. EFSA J.

[59] European Food Safety Authority (2012). Scientific opinion on dietary reference values for protein. EFSA J.

[60] Turck D, Bohn T, Castenmiller J (2022). Tolerable upper intake level for dietary sugars. EFSA J.

[61] Montejano Vallejo R, Schulz C-A, van de Locht K (2022). Associations of Adherence to a Dietary Index Based on the EAT-lancet reference diet with nutritional, anthropometric, and ecological sustainability parameters: results from the German DONALD Cohort Study. J Nutr.

[62] Oluwagbemigun K, Schnermann ME, Schmid M (2022). A prospective investigation into the association between the gut microbiome composition and cognitive performance among healthy young adults. Gut Pathog.

[63] Brachem C, Langenau J, Weinhold L (2020). Associations of BMI and body fat with urine metabolome in adolescents are sex-specific: a cross-sectional study. Metabolites.

[64] Brachem C, Oluwagbemigun K, Langenau J (2022). Exploring the association between habitual food intake and the urine and blood metabolome in adolescents and young adults: a cohort study. Mol Nutr Food Res.

[65] Brachem C, Weinhold L, Alexy U (2023). Replication and mediation of the association between the metabolome and clinical markers of metabolic health in an adolescent cohort study. Sci Rep.

[66] Muli S, Brachem C, Alexy U (2023). Exploring the association of physical activity with the plasma and urine metabolome in adolescents and young adults. Nutr Metab (Lond).

[67] Schnermann ME, Schulz C-A, Ludwig C (2022). A lifestyle score in childhood and adolescence was positively associated with subsequently measured fluid intelligence in the DONALD cohort study. Eur J Nutr.

[68] Remer T, Johner SA (2016). DONALD – ein Sensor für die Jodversorgung in Deutschland. Ernährungs Umschau.

[69] Remer T, Hua Y, Esche J (2022). The DONALD study as a longitudinal sensor of nutritional developments: iodine and salt intake over more than 30 years in German children. Eur J Nutr.

[70] Johner SA, Thamm M, Stehle P (2014). Interrelations between thyrotropin levels and iodine status in thyroid-healthy children. Thyroid.

[71] Roßbach S, Diederichs T, Nöthlings U (2018). Relevance of chronotype for eating patterns in adolescents. Chronobiol Int.

[72] Diederichs T, Roßbach S, Herder C (2016). Relevance of morning and evening energy and macronutrient intake during childhood for body composition in early adolescence. Nutrients.

[73] Wittmann M, Dinich J, Merrow M (2006). Social Jetlag: misalignment of biological and social time. Chronobiol Int.

[74] Perrar I, Schadow AM, Schmitting S (2020). Time and age trends in free sugar intake from food groups among children and adolescents between 1985 and 2016. Nutrients.

[75] WHO (2015). Guideline: sugars intake for adults and children.

[76] Hilbig A, Foterek K, Kersting M (2015). Home-made and commercial complementary meals in German infants: results of the DONALD study. J Hum Nutr Diet.

[77] Dietz WH (1994). Critical periods in childhood for the development of obesity. Am J Clin Nutr.

[78] Lanigan J, Singhal A (2009). Early nutrition and long-term health: a practical approach. Proc Nutr Soc.

[79] Ahrens W, Siani A, Adan R (2017). Cohort Profile: the transition from childhood to adolescence in European children-how I.Family extends the IDEFICS cohort. Int J Epidemiol.

[80] Wagner MO, Bös K, Jekauc D (2014). Cohort profile: the Motorik-Modul Longitudinal Study: physical fitness and physical activity as determinants of health development in German children and adolescents. Int J Epidemiol.

[81] Fraser A, Macdonald-Wallis C, Tilling K (2013). Cohort Profile: the avon longitudinal study of parents and children: ALSPAC mothers cohort. Int J Epidemiol.

[82] Kooijman MN, Kruithof CJ, van Duijn CM (2016). The Generation R Study: design and cohort update 2017. Eur J Epidemiol.

[83] Pickoff AS, Berenson GS, Schlant RC (1995). Introduction to the symposium celebrating the Bogalusa Heart Study. Am J Med Sci.

[84] Holtermann B, Dulon M (2001). Stillen in der Geburtsklinik und im 1. Lebensjahr: Ergebnisse der bundesweiten SuSe-Studie (Breast feeding in the maternity clinic and in the 1st year of life: results of the comprehensive country-wide SuSe Study). Kinderkrankenschwester.

[85] Mensink GB, Haftenberger M, Thamm M (2001). Original Communications-Validity of DISHES 98, a computerised dietary history interview: energy and macronutrient intake. Eur J Clin Nutr.

[86] Mensink GBM (2007) Die aktuelle Nährstoffversorgung von Kindern und Jugendlichen in Deutschland. Ernährungsumschau 636–646

[87] Robert Koch-Institut (2018) Übergewicht und Adipositas im Kindes- und Jugendalter in Deutschland – Querschnittergebnisse aus KiGGS Welle 2 und Trends. RKI-Bib1 (Robert Koch-Institut)

