# Supplementary material for: Cohort profile update–overview of over 35 years of research in the Dortmund Nutritional and Anthropometric Longitudinally Designed (DONALD) study
Source: Eur J Nutr. 2023 Dec 27;63(3):727–40. doi: 10.1007/s00394-023-03290-x (PMC10948456; doi:10.1007/s00394-023-03290-x)
Supplement: Supplementary file 1 — Supplementary file1 (DOCX 28 KB) [file 394_2023_3290_MOESM1_ESM.docx]

**Supplementary Material**

**Table S1:** Overview of additional modules in the DONALD study

| **Additional module** | **Year of data collection** | **Age of the participants** | **n** |
| --- | --- | --- | --- |
| Stool sample collection | 2017-2018 | ≥ 18 years | 205 |
| Personality-related characteristics | 2016-2020 | 5-6 years (Delay of Gratification, until June 2016 also 4 year olds)  7-9 years (Delay Discounting and Social Preference) | 126  168 |
| Genetics | 2015-2022 | < 18 (saliva samples)  ≥ 18 (blood samples) | 610 |
| Fluid intelligence | 2017-2018 | 5 - <8.5 years (CFT-1 R)  ≥ 8.5 (CFT-20 R) | 327 |
| Accelerometer | 2020-2022 | ≥ 6 years | 163 |
| Bone Density | 1998-1999 | 6-18 years | 371 |

**Figure S1:** Flowchart of the DONALD study population including the DONALD cohort and the reduced programme as well as the respective number of observations.
